# Comparison of the Psychopharmacological Effects of Tiletamine and Ketamine in Rodents

**DOI:** 10.1007/s12640-017-9759-0

**Published:** 2017-06-02

**Authors:** Piotr Popik, Małgorzata Hołuj, Tomasz Kos, Gabriel Nowak, Tadeusz Librowski, Kinga Sałat

**Affiliations:** 1 Faculty of Health Sciences, Jagiellonian University Medical College, 12 Michałowskiego Street, 31-126 Kraków, Poland; 2 Institute of Pharmacology, Polish Academy of Sciences, 12 Smętna Street, 31-343 Kraków, Poland; 3 Faculty of Pharmacy, Jagiellonian University Medical College, 9 Medyczna Street, 30-688 Kraków, Poland

**Keywords:** Ketamine, Tiletamine, Antidepressant-like effects, NMDA receptor

## Abstract

The glutamate *N*-methyl-d-aspartate (NMDA) receptor antagonist ketamine (KET) produces rapid and sustained antidepressant effects in patients. Tiletamine (TIL; 2-ethylamino-2-thiophen-2-yl-cyclohexan-1-one) is another uncompetitive NMDA receptor antagonist, used in a medical (veterinary) setting as an anesthetic tranquilizer. Here, we compared the behavioral actions of KET and TIL in a variety of tests, focusing on antidepressant-like and dissociative-like effects in mice and rats. The minimum effective doses of KET and TIL were 10 mg/kg to reduce mouse forced swim test immobility and 15 mg/kg to reduce marble-burying behavior. However, at similar doses, both compounds diminished locomotor activity and disturbed learning processes in the mouse passive avoidance test and the rat novel object recognition test. KET and TIL also reduced social behavior and accompanying 50-kHz “happy” ultrasonic vocalizations (USVs) in rats. TIL (5–15 mg/kg) displayed additional anxiolytic-like effects in the four-plate test. Neither KET nor TIL affected pain response in the hot plate test. Examination of the “side effects” revealed that only at the highest doses investigated did both compounds produce motor deficits in the rotarod test in mice. While KET produced behavioral effects at doses comparable between species, in the rats, TIL was ~10 times more potent than in the mice. In summary, antidepressant-like properties of both KET and TIL are similar, as are their adverse effect liabilities. We suggest that TIL could be an alternative to KET as an antidepressant with an additional anxiolytic-like profile.

## Introduction

The rapid and sustained antidepressant effects of ketamine (KET) (Zarate et al. 2006a; Berman et al. 2000) belong to the most intriguing discoveries and most often discussed topics in the current pharmacotherapy of major depression disorder (MDD). However, the mechanism of this unique action remains controversial and unexplained (Schatzberg 2014). KET acts primarily as a use-dependent antagonist at glutamate NMDA receptors (NMDARs; for a broader panel of other CNS targets, see Salat et al. (2015c)); thus, among several mechanisms related to its antidepressant effects, the inhibition of NMDARs is the most important. The observation that NMDAR antagonists display antidepressant-like properties originates from the mid-90s and was first proposed by Skolnick and coworkers at NIH (Skolnick et al. 1996; Trullas and Skolnick 1990). However, other than KET, clinically used uncompetitive NMDAR antagonists such as memantine failed in clinical trials as antidepressants (Zarate et al. 2006b). Another hypothesis involves KET metabolites (Domino 2010), acting perhaps on other targets, likely involving AMPA receptors (Zanos et al. 2016). This area is also controversial, because only norketamine (demonstrating decent affinity at NMDARs, i.e., producing a 56% inhibition of PCP binding sites at 10 μM) reduced immobility in the mouse forced swim test (FST), while dehydronorketamine exhibited no antidepressant-like actions in mice and no substantial activity (12%) at NMDARs (Salat et al. 2015c).

KET (Krystal et al. 1994), like other uncompetitive NMDAR antagonists (Morris and Wallach 2014), produces profound PCP-like (Luby 1959) psychotomimetic effects in humans including dissociative states, alterations in perception, and schizophrenia-like positive and negative symptoms. From this perspective, the failure of memantine to produce clinical antidepressant effects (Zarate et al. 2006b) was likely due either to insufficient dosing and/or to its micromolar affinity at NMDARs which is lower than that of KET (Kornhuber et al. 1991), and thus, the psychotomimetic effects are also lower than those of KET. This hypothesis is at least partly supported both by clinical findings showing that another NR2B subunit-selective NMDAR antagonist, CP-101,606 (traxoprodil), also produced dissociative effects on top of antidepressant actions in patients with MDD (Preskorn et al. 2008) and by studies on KET which revealed that the degree of dissociative symptoms experienced during KET infusions robustly correlated with the degree of reported depression rating scale improvement (Luckenbaugh et al. 2014). On the other hand, GLYX-13 (rapastinel), a novel NMDAR glycine-site functional partial agonist, produced an antidepressant effect without psychotomimetic side effects typical for NMDAR antagonists (Burgdorf et al. 2013; Moskal et al. 2014), which suggests that dissociative effects should not be regarded as the only mechanism underlying antidepressant activity observed in clinical settings.

While clinical trials are now being conducted with several other than KET ligands of NMDARs (AXS-05, AVP-786, Esketamine, CERC-301, GLYX-13; NRX-1074, AV-101; (Murrough 2016)), in the present study, we focused on tiletamine (TIL; 2-ethylamino-2-thiophen-2-yl-cyclohexan-1-one), which is structurally and functionally similar to KET. TIL is a use-dependent NMDAR antagonist (Rao et al. 1991; ffRench-Mullen et al. 1987) and an anesthetic tranquilizer used in veterinary medicine as a component of the product named Telazol® or Zoletil® (tiletamine/zolazepam).

TIL was developed by Parke-Davis in the 1960s as an alternative to KET and phencyclidine (PCP) (Chen et al. 1969). While it is currently contraindicated in patients, anecdotal reports indicate its KET-like or PCP-like properties. For instance, at Erowid Experience Vaults (https://www.erowid.org/pharms/tiletamine/), anonymous psychonauts (individuals who use mind-altered states to explore perceptual and spiritual phenomena) have reported profound dissociative, psychotomimetic, and amnestic properties of Telazol, much stronger than those of KET. Telazol has been reported to produce less cardiovascular depression (c.f., Quail et al. 2001) than KET, which also shows urinary tract/bladder toxicity (c.f. Morris and Wallach 2014). The unique pharmacology of TIL regarding dopaminergic system was studied by Rao et al. (1991), who reported that in contrast to MK-801, KET, and PCP, TIL did not increase pyriform cortex DOPAC levels (i.e., did not increase DA metabolism and/or release), suggesting some unique action not shared by other NMDAR antagonists.

Other CNS-related properties of TIL are much less explored as compared to those of KET, and therefore knowledge of its psychopharmacological profile is limited. In particular, little is known about its potential antidepressant activity. Hence, the main aim of this study was to compare the pharmacological properties of TIL to those of KET in rodent models. Because of its clinical (veterinary) use and established safety, its psychoactive properties resembling KET’s dissociative states, and somewhat different from KET pharmacology, we compared the behavioral properties of TIL to those of KET in rodent tests of depression, anxiety, cognition, and negative-like symptoms of psychoses. To interpret the results from in vivo tests properly, we also investigated the influence of TIL and KET on animals’ locomotor activity, pain threshold, and potential motor deficits.

## Materials and Methods

### Animals

The study included adult male albino Swiss (CD-1) mice weighing 18–22 g (Animal Breeding Farm of the Jagiellonian University Faculty of Pharmacy, Poland) and male Sprague-Dawley rats (Charles River, Germany), weighing 200–250 g (novel object recognition test (NORT)) or 125–150 g (social interaction test) on arrival.

Mice were kept in groups of 10 in standard plastic cages and housed under controlled conditions (room temperature of 22 ± 2 °C, light/dark (12:12) cycle, lights on at 8.00 a.m., humidity 50–60%, and free access to food and water). Rats were housed in a temperature- (21 ± 1 °C) and humidity-controlled (40–50%) colony room under a 12:12-h light/dark cycle (lights on at 06:00 a.m.).

All experiments, except for sucrose preference tests, were performed between 9 a.m. and 3 p.m. All procedures were approved by the respective local ethics committees, and the treatment of animals was in full accordance with ethical standards laid down in respective Polish and EU regulations (Directive No. 86/609/EEC).

### Chemicals

TIL hydrochloride (MedChemExpress, NJ, USA) was prepared in 0.9% saline solution. KET (aqueous solution (115.34 mg/ml), Vetoquinol Biowet, Gorzów Wielkopolski, Poland) was diluted in distilled water to the appropriate concentrations. Drugs were administered intraperitoneally at a volume of 10 ml/kg (mice) and 1 ml/kg (rats), 30 min before the behavioral tests. The doses of KET used in the present research were chosen based on our previous studies (Potasiewicz et al. 2017; Salat et al. 2015c) and available literature data (Eskelund et al. 2017; Koike et al. 2011; Zhu et al. 2017). Since there is a limited amount of data regarding effective doses of TIL in rodents (Gargiulo et al. 2012; Su et al. 2017), we conducted preliminary dose-response studies (data not shown) to establish the starting dose of TIL (5 mg/kg in mice and 0.5 mg/kg in rats).

### Behavioral Procedures

#### Antidepressant-Like: Mouse Forced Swim Test

This experiment was carried out according to the method originally described by Porsolt et al. (1977) with some minor modifications (Salat et al. 2015c). Mice were dropped individually into glass cylinders (height = 25 cm, diameter = 10 cm) filled with water to a height of 10 cm and maintained at 23–25 °C. The animals were left in the cylinder for 6 min. The total duration of immobility was recorded during the final 4 min of the whole 6-min testing period. Mice were judged to be immobile when they remained floating passively in the water, making only small movements to keep their heads above the water surface.

#### Antidepressant-Like (Anhedonia): Mouse Sucrose Preference Test

Prior to the experiment, mice were placed into separate cages. Two pre-weighed bottles, one containing tap water and the other containing 1% sucrose solution, were placed on each cage. The bottle order (left-right placement of water vs. sucrose bottles) was counterbalanced among mice in each group. In this test, the mice were given a 48-h free choice between the two bottles. At the beginning and the end of the test, the bottles were weighed and consumption was calculated. The test was begun with the onset of the dark (active) phase of the animals’ cycle. The position of the bottles in the cage was switched every 12 h. Before the test, no food or water deprivation was applied (Strekalova et al. 2004). The preference for sucrose was calculated as a percentage of consumed sucrose solution in terms of the total amount of liquid drunk.

#### Anxiety: Mouse Four-Plate Test

The four-plate apparatus (Bioseb, France) consists of a cage (25 cm × 18 cm × 16 cm) that is floored with four rectangular metal plates (11 cm × 8 cm). The plates are separated from one another by a gap of 4 mm, and they are connected to an electroshock generator. The test was performed according to Bourin et al. (2005). After the habituation period (15 s), each mouse was subjected to an electric shock (0.8 mA, 0.5 s) when crossing from one plate to another (two limbs on one plate and two on another). The number of punished crossings was counted during 60 s.

#### Anxiety (Obsessive-Compulsive Behavior), Depression, Irritability and Impulsivity: Mouse Marble-Burying Test

The test was performed according to a method described by Broekkamp et al. (1986), with some minor modifications. Briefly, the mice were placed individually into plastic cages identical to their home cages. The cages contained a 5-cm layer of sawdust and 20 black glass marbles (1.5 cm diameter), which were gently placed in the cage, equidistant in a 4 × 5 arrangement. After a 30-min testing period, the mice were removed from the cages and the number of marbles at least 2/3 buried was counted.

#### Cognition: Mouse Passive Avoidance Task

The test was conducted according to Salat et al. (2015b) using a passive avoidance apparatus (Panlab Harvard Apparatus, Spain) consisting of a large white-painted illuminated compartment (26 × 26 × 34 cm) and a small black-painted dark compartment (13 × 7.5 × 7.5 cm) separated from each other by a guillotine gate. Mice underwent two separate trials, an acquisition trial (conditioning phase) and a retention trial (testing phase), conducted 24 h after the acquisition trial. For the acquisition trial, each mouse was initially placed for 30 s in the light compartment (exploration period; guillotine gate is closed). At the end of the exploration period, the guillotine door (5 × 5 cm) was opened and the time elapsed before entering the black chamber was recorded. As soon as the mouse entered the dark compartment, the door was automatically closed and an electrical shock (current intensity = 0.2 mA, duration = 2 s) was delivered through the grid floor. For the retention trial, the mice were placed in the illuminated white compartment again, and the latency time between door opening and entry into the dark compartment was recorded for each mouse up to 180 s (cutoff latency).

#### Cognition: Rat Novel Object Recognition Test

The protocol described earlier (Nikiforuk et al. 2013a) was adapted from the original work of Ennaceur and Delacour (1988). At least 1 h before the start of the experiment, rats were transferred to the experimental room for acclimation. Animals were tested in a dimly lit (25 lx) open field apparatus made of a dull gray plastic (66 × 56 × 30 cm). After each measurement, the floor was cleaned and dried. The procedure consisted of a 5–min habituation to the arena without any objects, 24 h before the test. The testing comprised two trials, separated by an inter-trial interval (ITI) of 1 h. During the first (familiarization, T1) test period, two identical objects (A1 and A2) were presented in opposite corners of the arena, approximately 10 cm from the walls. Following T1, the objects were cleaned with water containing a dishwashing agent and dried. In the second trial (recognition, T2), one of the objects was replaced by a novel one (A = familiar and B = novel). Both trials lasted for 3 min. After T1, animals were returned to their home cages. The objects used were a 250-ml glass beaker (diameter of 8 cm, height of 14 cm) and a 250-ml plastic bottle (6 × 6 × 13 cm). The location of the novel object in T2 was randomly assigned for each rat. Exploration of an object was defined as rats looking, licking, sniffing, or touching the object but not leaning against or standing or sitting on the object. Exploration time of the objects was measured using the Any-maze® tracking system (Stoelting Co., IL, USA). Based on the exploration time (*E*) of two objects, a discrimination index was calculated in accordance with formula DI = (EB − EA) / (EA + EB), where EA is defined as the time spent exploring the familiar object and EB is the time spent exploring the novel object, respectively.

#### Negative Symptoms of Schizophrenia-Like Measure: Rat Social Behavior

The experiments were conducted in an open field arena (length × width × height = 57 × 67 × 30 cm) made of black Plexiglas. The arena was dimly illuminated with an indirect light of 18 lx. The behavior of the rats was recorded using two cameras placed above the arena and connected to a Noldus MPEG recorder 2.1. An experimenter blind to the treatment conditions analyzed the videos off-line using Noldus Observer® XT, version 10.5. The rats were individually housed for 5 days prior to the start of the procedure. The animals were subsequently handled and weighed, and the backsides of one half of the animals were dyed with a gentian violet (2% methylrosanilinium chloride) solution. On the test day (the sixth day of social isolation), to reduce aggressive and territorial behaviors and to increase the level of social behavior, two unfamiliar rats of matched body weight (±5 g) were placed in the open field arena, and their behaviors were recorded for 10 min. The social interaction time was measured for each rat separately. The following active social behaviors were scored: sniffing (the rat sniffs the body of the conspecific), anogenital sniffing (the rat sniffs the anogenital region of the conspecific), social grooming (the rat licks and chews the fur of the conspecific), following (the rat moves toward and follows the other rat), mounting (the rat stands on the back of the conspecific), and climbing (the rat climbs over the back of the conspecific) (Holuj et al. 2015). No overt aggressive behaviors (such as biting, kicking, boxing, and threatening behavior) were observed in control animals or after treatment with KET or TIL. As the mean total time of aggressive behaviors was <3% of the session duration, aggression was not included in the analysis. The time of active social behaviors was summed to yield a total score. As both animals in a pair yielded approximately equal scores (for either total time spent in social interactions or separate social behaviors), social interaction time was expressed as a summed score for each pair of animals.

In addition, we also measured the number of 50-kHz ultrasonic vocalizations (USVs) that accompany rat social interactions and reflect a positive effect. This was done as described earlier (Nikiforuk et al. 2013b).

#### Mouse Locomotor Activity

The locomotor activity test was performed as previously described (Salat et al. 2015c) using activity cages (40 cm × 40 cm × 30 cm) supplied with I.R. beam emitters (Activity Cage 7441, Ugo Basile, Italy) connected to a counter for the recording of light-beam interrupts. The animals’ movements (i.e., the number of light-beam crossings) were counted during the next 30 min of the test in 10-min time epochs.

#### Analgesia: Mouse Hot Plate Test

The hot plate apparatus (Hot/Cold Plate, Bioseb, France) consists of an electrically heated surface and it is equipped with a temperature controller that keeps the temperature constant at 55–56 °C. The test was performed as previously described (Salat et al. 2015a). One day before the experiment, the animals were tested for their pain sensitivity threshold (baseline latency). For further pain tests, only mice with baseline latencies ≤30 s were selected. The latency time to pain reaction (licking hind paws or jumping) was measured as the indicative of nociception. The cutoff time was established (60 s) and animals that did not respond within 60 s were removed from the hot plate apparatus and assigned a score of 60 s.

#### Motor Coordination: Mouse Rotarod Test

Before the test, mice were trained daily for three consecutive days on a rotarod apparatus (May Commat RR0711, Turkey; rod diameter = 2 cm) that was rotating at a fixed speed of 18 rpm. In each session, the mice were placed on the rotating rod for 3 min with an unlimited number of trials. The proper experiment was performed 24 h after the last training session with the apparatus revolving at 6 or 24 rpm. Motor impairments were defined as the inability to remain on the rotarod apparatus for 1 min, and these were expressed as the mean time spent on the rotarod (Salat et al. 2015a).

#### Statistics

Data were analyzed using one-way and/or two-way ANOVA (IBM/SPSS 21 for Windows) with Dunnett’s post hoc test. The alpha value was set at *P* < 0.05. The homogeneity of variance was measured with Levene’s test.

## Results

### Antidepressant-Like: Mouse Forced Swim Test

**Fig. 1:**
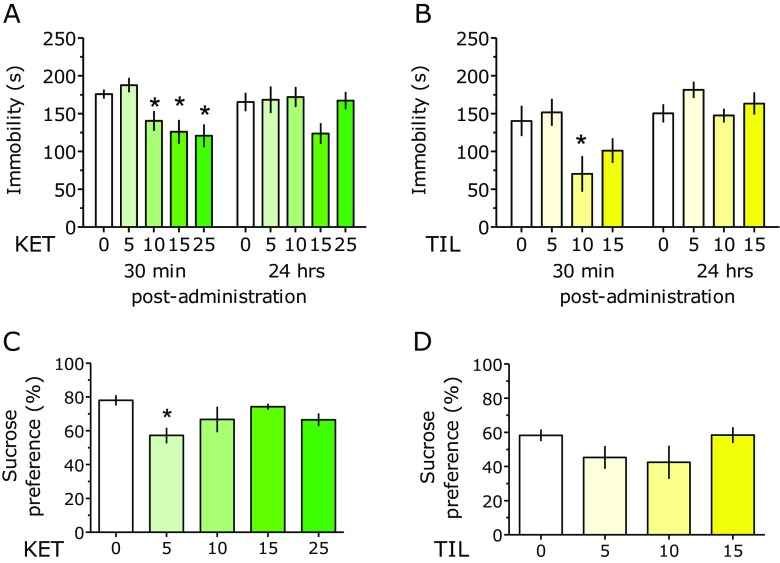
Effect of ketamine (*KET*) and tiletamine (*TIL*) on duration of immobility in the forced swim test (**a** and **b**, respectively) and on preference for sucrose (1%) over water (**c** and **d**, respectively). Results are shown as mean ± SEM. *N* = 7–8 mice per treatment. Symbols: *P < 0.05 vs. vehicle-treated mice

In the FST, two-way ANOVA demonstrated an overall effect of treatment with KET (*F*(4, 51) = 4.64; *P* < 0.01). Time did not affect the results (*F*(1, 51) = 1.38) and drug × time interaction was also insignificant (*F*(4, 51) = 2.44). For the sake of curiosity, separate one-way ANOVAs were calculated on 30-min post-treatment and 24-h post-treatment times, which showed significant effects of the treatment at 30 min (*F*(4, 51) = 5.73; *P* < 0.001) but not at 24-h post-administration (*F*(4, 51) = 2.19). Insignificant effects of KET 24-h post-administration could have masked an apparent effect of 30-min post-administration; indeed, at that time, KET reduced immobility at doses of 10, 15, and 25 mg/kg (Fig. 1a).

For TIL, two-way ANOVA showed the following values: drug effect (*F*(3, 28) = 3.41; *P* < 0.05), time effect (*F*(1, 28) = 21.91; *P* < 0.001), and drug × time interaction (*F*(3, 28) = 2.52). Again, separate one-way ANOVAs were calculated on two post-treatment times, which showed significant effects of treatment at 30 min (*F*(3, 28) = 3.62; *P* < 0.05) but not for the 24-h post-administration (*F*(3, 28) = 1.70). Again, insignificant effects of TIL 24-h post-administration could have masked an apparent effect of 30-min post-administration; indeed, at that time, TIL reduced immobility at the dose of 10 mg/kg (Fig. 1b).

### Antidepressant-Like (Anhedonia): Mouse Sucrose Preference Test

In the sucrose preference assay, a significant effect of KET on sucrose preference was demonstrated (*F*(4, 35) = 3.083, *P* < 0.05; Fig. 1c), while TIL displayed no activity (*F*(3, 35) = 1.59; Fig. 1d).

### Anxiety: Mouse Four-Plate Test

**Fig. 2:**
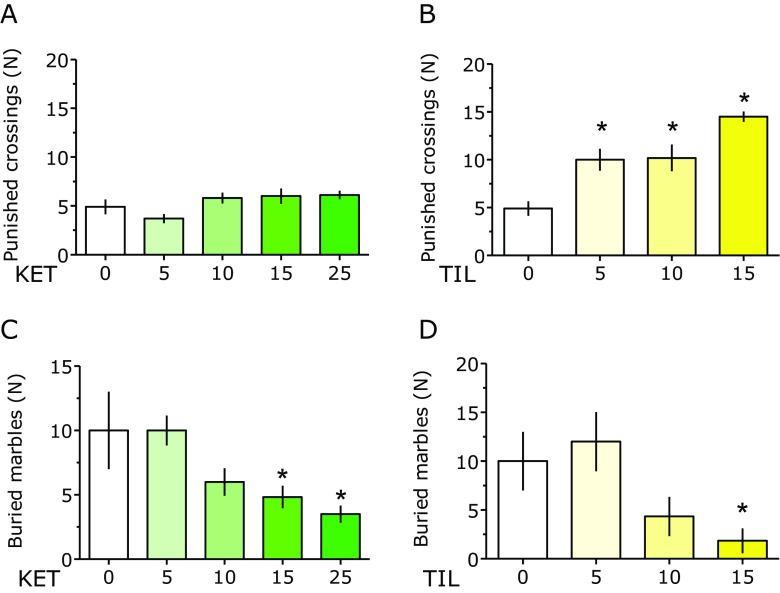
Effect of ketamine (*KET*) and tiletamine (*TIL*) on the number of punished crossings in the four-plate test (**a** and **b**, respectively) and number of buried marbles in the marble-burying test (**c** and **d**, respectively). Results are shown as mean and SEM. *N* = 6–10 mice per treatment. Symbols: *P < 0.05 vs. vehicle-treated mice

KET and TIL significantly affected the number of punished crossings: *F*(4, 43) = 2.59 and *P* < 0.05 and *F*(3, 34) = 13.29 and *P* < 0.001, respectively. While TIL at doses of 5–15 mg/kg significantly increased the number of crossings (Fig. 2b), none of the KET doses significantly affected the number of punished crossings (Fig. 2a).

### Anxiety (Obsessive-Compulsive Behavior), Depression, Irritability, and Impulsivity: Mouse Marble-Burying Test

Statistical analyses showed the following ANOVA values: *F*(4, 25) = 3.53 and *P* < 0.05 and *F*(3, 20) = 3.79 and *P* < 0.05 for KET and TIL, respectively. KET significantly reduced the number of buried marbles at the doses of 15–25 mg/kg (Fig. 2c); for TIL, only the dose of 15 mg/kg exerted a statistically significant effect (Fig. 2d).

### Cognition: Mouse Passive Avoidance Task

In the passive avoidance task, two-way ANOVA demonstrated an overall effect of treatment with KET (*F*(4, 45) = 2.91; *P* < 0.05). Time affected the results significantly (*F*(1, 45) = 102.95; *P* < 0.001) and drug × time interaction was also significant (*F*(4, 45) = 3.21; *P* < 0.05). In the acquisition trial, none of the KET doses affected entry latency in comparison with the vehicle. However, in the retention trial, KET at doses 5 and 10 (but not 15 or 25) mg/kg significantly reduced the latency to enter the dark compartment as compared with the vehicle, suggesting cognitive impairment produced by relatively lower doses.

**Fig. 3:**
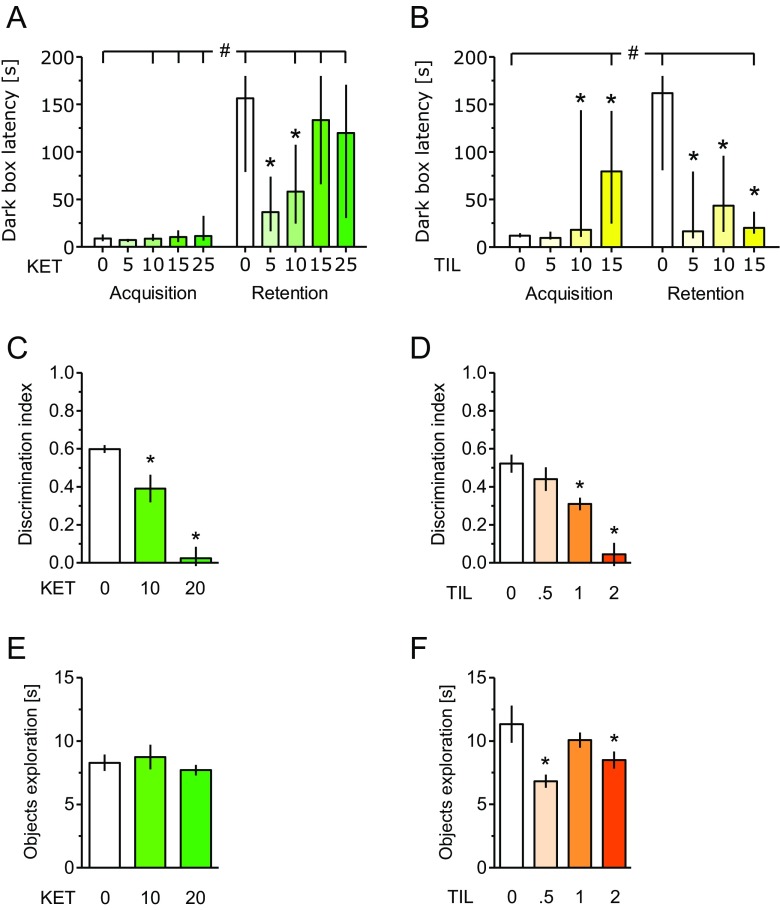
In the passive avoidance task in mice, ketamine (*KET*; **a**) and tiletamine (*TIL*; **b**) produced learning deficits in that they shortened the step-trough latencies to enter the dark compartment at the retention trial. For the vehicle and all doses of KET except for 5 mg/kg, retention latencies were longer than respective acquisition latencies (*number sign*), suggesting an unimpaired learning except for the only KET dose of 5 mg/kg (**a**). In the TIL experiment (**b**), only one dose (15 mg/kg) resulted in shorter retention than acquisition latency (*number sign*), suggesting learning deficit produced by this dose. In rat’s NORT, both compounds produced cognitive impairment (**c**, **d**), and TIL reduced object exploration (**f**). Data for passive avoidance are shown as median and interquartile range, and the rat NORT data as mean and SEM. *N* = 10 mice and 7–10 rats per treatment. Symbols: *P < 0.05 vs. respective vehicle control; #*P* < 0.05 vs. acquisition trial

In the passive avoidance test, increased latency to reenter the dark box serves as an index of learning. When latencies at acquisition and retention trials were compared within a given treatment, for the vehicle and all doses of KET, except for 5 mg/kg, retention latencies were longer than respective acquisition latencies, suggesting somewhat unimpaired learning except for only a KET dose of 5 mg/kg (Fig. 3a).

For TIL, two-way ANOVA showed the following values: drug effect (*F*(3, 36) = 2.68; *P* = 0.06), time effect (*F*(1, 36) = 5.54; *P* < 0.05), and drug × time interaction (*F*(3, 36) = 18.83; *P* < 0.001. In the acquisition trial, TIL doses of 10 and 15 mg/kg appeared to increase entry latencies, suggesting potential sedative action or motor impairment. In the retention trial, TIL at doses of 5–15 mg/kg reduced latencies to enter the dark compartment, suggesting cognitive impairment.

When latencies in acquisition and retention trials were compared within a given treatment, only for the vehicle-treated group was retention latency longer than respective acquisition latency. Only one dose of TIL (15 mg/kg) resulted in a shorter retention than acquisition latency, suggesting learning deficit (Fig. 3b).

### Cognition: Rat Novel Object Recognition Test

As shown in Fig. 3c, d, KET (10–20 mg/kg) and TIL (1–2 mg/kg) disturbed NORT at relatively short ITI of 1 h: *F*(2, 26) = 26.86 and *P* < 0.001 and *F*(3, 28) = 16.95 and *P* < 0.001, respectively.

In the same test, we measured the total time of either the exploration of objects in the acquisition (T1) trial, purportedly reflecting rats’ propensity to explore novel objects, or sedation. While KET (Fig. 3e) did not affect this measure (*F*(2, 26) = 0.49), TIL (0.5 and 2 mg/kg; Fig. 3f) reduced it compared to the vehicle (*F*(3, 28) = 4.82, *P* < 0.01).

### Negative Symptoms of Schizophrenia-Like Measure: Rat Social Behavior

**Fig. 4:**
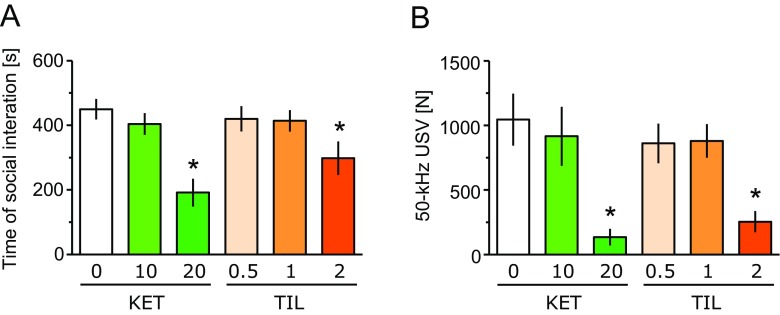
Ketamine (*KET*) and tiletamine (*TIL*) reduce the time of social interactions (**a**) and the number of “happy” 50-kHz ultrasonic calls (**b**) recorded during rat social encounter. Data represent mean ± SEM. *N* = 5 pairs of rats per treatment. Symbols: *P* < 0.05 vs. vehicle-treated rats

Administration of KET (20 mg/kg) and TIL (2 mg/kg) reduced total social interaction time compared to the vehicle-treated animals (one-way ANOVA: *F*(5, 24) = 6.29; *P* < 0.001; Fig. 4a) and the number of USVs emitted by the rats during social encounters (*F*(5, 24) = 6.17; *P* < 0.001; Fig. 4b).

### Mouse Locomotor Activity, Analgesia, and Motor Coordination

**Fig. 5:**
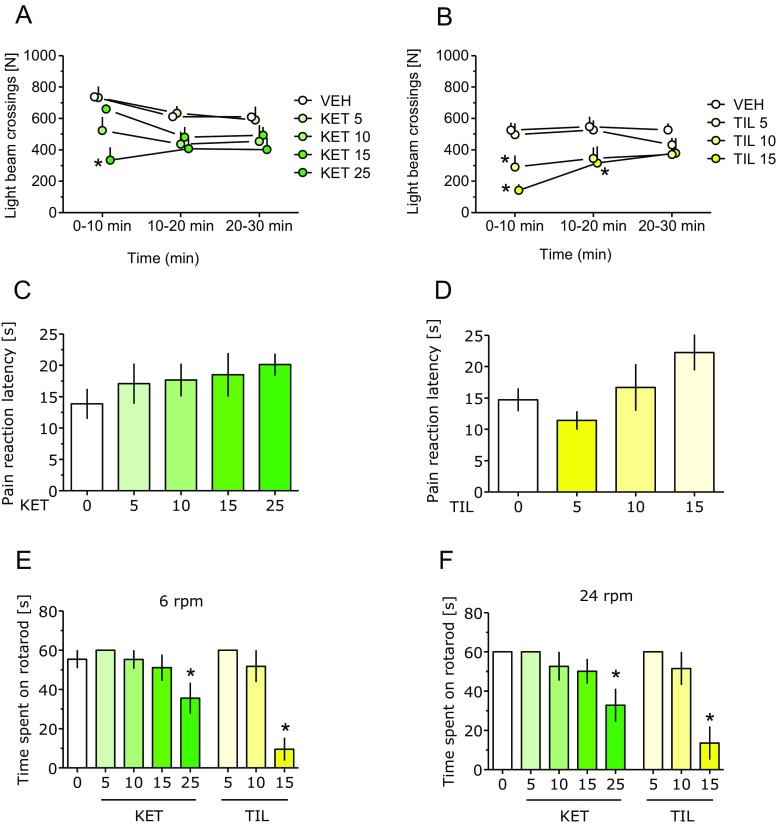
Effect of ketamine (*KET*) and tiletamine (*TIL*) on locomotor activity (**a** and **b**, respectively), analgesia in the hot plate test (**c** and **d**, respectively) and motor coordination in rotarod revolving at 6 and 24 rpm (**e** and **f**, respectively). Results are shown as mean and SEM. *N* = 8 (locomotor activity), 8 (KET hot plate), 10 (TIL hot plate), and 7 (rotarod) mice per treatment. Symbols: *P < 0.05 vs. vehicle-treated mice

An overall treatment effect of KET on locomotor activity was observed (*F*(4, 35) = 3.82; *P* < 0.05). Time affected the results significantly (*F*(2, 70) = 9.20; *P* < 0.001) and drug × time interaction was also significant (*F*(8, 70) = 2.27; *P* < 0.05; Fig. 5a). KET reduced activity at 25 (but not 5–15) mg/kg and only within the first measurement epoch, i.e., up to 10 min following administration. For TIL, statistical analysis showed the following ANOVA values: drug effect (*F*(3, 28) = 3.90; *P* < 0.05), time effect (*F*(2, 56) = 4.82; *P* < 0.05), and drug × time interaction (*F*(6, 56) = 3.55; *P* < 0.01; Fig. 5b). TIL at 10–15 mg/kg reduced activity at the beginning of the measurement; the dose of 15 mg/kg also reduced it up to 20 min following administration.

In the hot plate test, KET at doses of 5–25 mg/kg did not demonstrate analgesic properties (*F*(4, 35) = 0.70; Fig. 5c). While for TIL, ANOVA yielded significant treatment differences (*F*(3, 36) = 3.0; *P* < 0.05; Fig. 5d) and none of the doses produced significant alterations in pain reaction latency.

In the mouse rotarod test, the impact of KET and TIL on motor coordination was assessed at 6 and 24 rpm separately (Fig. 5e and f, respectively). For 6 rpm, ANOVA values were *F*(7, 48) = 9.36 and *P* < 0.001 and for 24 rpm *F*(7, 48) = 7.05 and *P* < 0.001. At both speeds, KET at 25 and TIL at 15 mg/kg reduced motor coordination.

## Discussion

The main goal of the present study was to assess potential antidepressant-like properties of TIL and compare them to those of KET. We also attempted to hypothesize as to which tests could be indicative or useful in elucidating KET’s enduring antidepressant-like effects. An unexpected finding of the present study was that while KET produced behavioral effects at doses comparable between species, in rats, TIL was ~10 times more potent. At present, we cannot offer an explanation for this finding; however, both compounds are antagonists at NMDARs, with KET affinity of 119–1000 nM (see (Salat et al. 2015c) and references therein). Our unpublished data (A. Siwek) revealed that TIL *K*_i_ at [^3^H]-MK-801 sites was 69 ± 14 nM (*N* = 3), which agrees with Rao et al. (1991) data (IC_50_ at [^3^H]-TCP labeled sites ~79 nM), suggesting that TIL is six to eight times more potent than KET at NMDARs.

The results of the present in vivo study are summarized in Table 1 that shows that while KET and TIL produced antidepressant-like action in the mouse FST and anti-obsessive-compulsive effect in marble-burying test, they also reduced locomotor activity and disturbed learning processes. The reduction of locomotor activity indicates the specific anti-immobility effect in the FST, because stimulant effects are regarded as unspecific. However, the antidepressant-like activity of TIL in FST was not stronger than that of KET, and TIL reduced immobility at only one - (mid-) dose, whereas KET was effective at doses 10–25 mg/kg. Moreover, investigating behaviorally naive mice, we noted no enduring antidepressant-like effects of KET and TIL in FST, which agrees with previous reports (Bechtholt-Gompf et al. 2011; Popik et al. 2008). This confirms that the “normal” mouse FST is not suitable and sensitive enough to detect persistent antidepressant-like effects of KET and that animal models of depression such as rat chronic mild stress (Papp et al. 2017) and mouse chronic social defeat stress and lipopolysaccharide-induced depression-like phenotypes (Yang et al. 2017) are more appropriate. The limitation of the present experiments was the lack of a time-course study.

**Table 1:** Summary of behavioral effects of ketamine (KET) and tiletamine (TIL) in mice and rats

Test	Species	KET active dose(s)	TIL active dose(s)	Figure
Immobility in FST (antidepressant-like)	Mouse	10–25 (↓)	10 (↓)	Figure 1a, b
Sucrose preference (antidepressant-like, anhedonia)	Mouse	10 (↓)	–	Figure 1c, d
Four-plate (anxiolytic-like)	Mouse	–	5–15 (↑)	Figure 2a, b
Marble-burying (anxiolytic-like, obsessive-compulsive behavior antidepressant-like, irritation, and/or perseveration)	Mouse	15–25 (↓)	15 (↓)	Figure 2c, d
Latency to enter dark compartment in passive avoidance (cognition)	Mouse	5,10 (↓)	5–15 (↓)	Figure 3a, b
Novel object recognition (cognition)	Rat	10–20 (↓)	1–2 (↓)	Figure 3c, d
Novel object recognition (exploration)	Rat	–	0.5, 2 (↓)	Figure 3e, f
Social interaction and 50-kHz USV emission (social withdrawal, negative-like symptoms of schizophrenia, psychotomimetic-like)	Rat	20 (↓)	2 (↓)	Figure 4a, b
Locomotor activity	Mouse	25 (↓)	10–15 (↓)	Figure 5a, b
Hot plate (antinociceptive action)	Mouse	–	–	Figure 5c, d
Motor coordination in rotarod	Mouse	25 (↓)	15 (↓)	Figure 5e, f

KET at the lowest dose tested (5 mg/kg) unexpectedly reduced sucrose preference, i.e., it augmented anhedonia, whereas the treatment with TIL did not influence sucrose intake at any of the doses used. The sucrose preference test is a reward-based assay used to detect anhedonia-like state in rodents (Strekalova et al. 2004; Papp et al. 2017). The results obtained for KET appear to contradict those reported by Papp et al. (2017) and Yang et al. (2017), who, however, investigated the effect of KET in animal models of depression, while we used naive mice. Also, in the Papp et al. study, KET did not affect sucrose intake in non-stressed controls (Papp et al. 2017).

Examination of dissociative-like effects revealed that both compounds disturbed social behavior and reduced 50-kHz USV emission in rats. Of note was the fact that in both assays for KET, this effect reached statistical significance at a dose 10-fold higher than that for TIL (20 vs. 2 mg/kg). Investigation of the “side effects” demonstrated that only at the highest doses did both compounds produce motor deficits in the rotarod test. In addition, neither KET nor TIL affected pain response in the hot plate test. This acute pain model was used as a control for the passive avoidance and four-plate tests, and it enabled the exclusion of potential false positive results in these two assays.

Using a preliminary assay based on the unconditioned fear model of anxiety, i.e., the four-plate test, we also investigated if KET or TIL could have anxiolytic-like properties in mice. This test revealed that TIL, in contrast to KET, possessed additional anxiolytic-like properties. These results should be taken with care, as we implemented only one behavioral test and further extended research is required to confirm this activity of TIL in other tests, such as the elevated plus maze which is based on the natural aversion of mice for open and elevated areas and on their natural spontaneous exploratory behavior in novel environments. Hayase et al. (2006) reported no effects of KET in the elevated plus maze test in ICR mice, while Silvestre et al. (1997) used three non-conflict tests (holeboard, social interaction, and elevated plus maze paradigms) and observed (a) decreased time spent in the active social interaction, (b) decreased percentage of time spent in open arms of the elevated plus maze, and (c) no significant effect on head dipping in the holeboard test. These authors suggested an anxiogenic-like effect of KET that contrasted with the effects produced by other uncompetitive NMDAR antagonists and resembled those described for stimulant drugs such as caffeine, cocaine, or amphetamine. While we used a different (four-plate) test, our data agree with the above, in that KET displays no anxiolytic-like actions. However, TIL increased the number of punished crossings in the four-plate test and this effect appeared specific, as this drug did not increase animals’ locomotor activity.

The marble-burying behavior, similarly to the four-plate test, comprises many kinds of domains related to anxiety, so it can be interpreted in various ways. Firstly, marble-burying has been suggested to reflect a form of impulsive behavior (Gyertyan 1995), and has even been regarded as a model of obsessive-compulsive disorder (Borsini et al. 2002; Njung'e and Handley 1991; Broekkamp et al. 1986; Li et al. 2006) in which the majority of antidepressants are effective in the attenuation of symptoms (reviewed by Borsini et al. 2002; Ammar et al. 2015). Acute administration of selective serotonin reuptake inhibitors, tricyclic antidepressants, selective noradrenaline reuptake inhibitors, and dual noradrenaline/serotonin reuptake inhibitors selectively and dose-dependently suppressed marble-burying behavior in mice (Schneider and Popik 2007; Marinova et al. 2017; Rodriguez et al. 2013). Secondly, the suppression of spontaneous burying of harmless objects by rodents is known to be sensitive to anxiolytic drugs rather than antipsychotics (Broekkamp et al. 1986; Njung'e and Handley 1991). Recently, a positive effect of memantine as an augmentation therapy for obsessive-compulsive disorder has been demonstrated (Marinova et al. 2017). KET is effective in patients with treatment-resistant depression, obsessive-compulsive disorder, and post-traumatic stress disorder (Glue et al. 2017; Rodriguez et al. 2013). This rapid anti-obsessive-compulsive effect achieved after a single intravenous dose of KET persisted for at least 1 week (Rodriguez et al. 2013). Our findings are in line with those mentioned above, as both KET and TIL significantly reduced marble-burying behavior at a comparable dose of 15 mg/kg. However, the analysis of both the four-plate test’s and marble-burying test’s results indicates the superiority of TIL over KET in anxiety-spectrum disorders.

The analysis of social behaviors of pairs of unfamiliar rats represents an ethologically valid approach for the preclinical assessment of social functions (Sams-Dodd 2013) and in some settings, not used in the present study (unfamiliar environment and high level of lights), serves to measure anxiety. NMDAR antagonists (Koros et al. 2007), including KET (Nikiforuk et al. 2013b), are capable of modeling negative-like symptoms of psychoses expressed as a social withdrawal. The present data are consistent with these findings, in that both KET and TIL reduced the time spent in active social interactions. In addition, we showed that both compounds reduced 50-kHz ultrasonic “happy” calls that accompany social behavior (Nikiforuk et al. 2013b). Such effects have been interpreted as being indicative for psychotomimetic actions, that is, hallucinations and delusions (Sams-Dodd 2013). In the context of enduring antidepressant actions of KET (Zarate et al. 2006a; Berman et al. 2000), we do not view these data as “undesired side” effects, particularly in light of the reports presented in the “Introduction” section (Griffiths et al. 2016).

Both the passive avoidance test in mice and the novel object recognition test in rats demonstrated amnestic actions of KET and TIL. While NMDAR antagonists impaired cognitive processes in naive subjects, KET displays pro-cognitive effects in stressed or “depressed” rats (Nikiforuk and Popik 2014; Papp et al. 2017). Nonetheless, these data further suggest that both compounds could have dissociative-like effects reflecting disturbed attention of animals.

Using the hot plate test (i.e., the thermally induced pain model), we examined whether purported analgesic properties of KET or TIL could have contributed to the amnestic effects observed in the passive avoidance task and anxiolytic-like action in the four-plate test. However, the present data agree with earlier reports (Plesan et al. 1998) and demonstrate no changes in heat pain thresholds after treatment with KET and TIL.

In summary, antidepressant-like properties of both KET and TIL, as well as their adverse effect liabilities, are similar. TIL has an additional anxiolytic-like profile. The present data demonstrate the usefulness of animal research in finding the dissociative-like states in preclinical settings purportedly necessary for the enduring antidepressant effects of noncompetitive NMDAR antagonists.
